# A Methodology Based on Pulse-Velocity Measurements to Quantify the Chemical Degradation Levels in Thin Mortar Specimens

**DOI:** 10.1007/s10921-018-0534-9

**Published:** 2018-10-20

**Authors:** Hector Hernandez Delgadillo, Benoit Kern, Richard Loendersloot, Doekle Yntema, Remko Akkerman

**Affiliations:** 1 Faculty of Engineering Technology (ET), University of Twente, Drienerlolaan 5, 7522 NB Enschede, The Netherlands; 2 Smart Water Grids Theme, Wetsus European Centre of Excellence for Sustainable Water Technology, Oostergoweg 9, 8911 MA Leeuwarden, The Netherlands; 3 ENSCMU – École Nationale Supérieure de Chimie de Mulhouse, 3 rue Alfred Werner, 68093 Mulhouse Cedex, France

**Keywords:** Ultrasonic testing, Pulse velocity, Mortar specimens, Degraded layer thickness

## Abstract

In this research, ultrasonic pulse echo measurements are used to quantify through thickness chemical degradation in thin mortar specimens. The degradation level is predicted using the time of travel of the acoustic wave through the thickness of the structure. The front and back wall interaction reflections are used to obtain additional information from very early stage degradation. The pulse-velocity of sound waves as a function of the thickness of the layers within the structure is described. With knowledge of the pulse-velocity in pristine and fully degraded conditions, it is possible to determine the complete range of degradation length over the layer thickness. The method is applicable for leaching of calcium and acidic attack. The acoustic measurements were verified with destructive testing. The correlation between the acoustic and non-acoustic experiments agree with the described pulse-velocity and degraded depth function. The method based on ultrasonic measurements can be implemented in other thin-layered structures.

## Introduction

The installation of a large part of the drinking water network in many countries was done in the mid-twentieth century. Nearly 30% of the Dutch water mains network is made of cementitious materials. Depletion of material initiated after the pipes were laid underground, so up until now, these assets have been under continuous deterioration. Deterioration leads to a decrease in load capacity and higher chance of malfunction. An inspection system capable of detecting and quantifying the amount of degradation within cement-based drinking water pipes will enhance the maintenance of the infrastructure and potentially avoid pipe failure.

The degradation mechanisms inherent in cementitious drinking water pipes are: leaching of calcium, acidic attack, carbonation, biodegradation and sulphate attack [1]. Deterioration of these cementitious pipes primarily occurs due to leaching of calcium. On the contrary, sulphate attack and acidic attack are less likely to occur. Yet, it has been shown that the generation of a layer of bacteria (biofouling) in the inner surface of the water distribution pipes will increase the acidity level [2, 3]. Deterioration in the outer surface of the pipe can occur due to mixed degradation mechanisms [1]. Furthermore, internal porosity in the structure leads to more complex interaction between inner and outer degraded layers [1].

Carbonation of hydrated products mainly is due to the percolation of ${\text{CO}}_{2}$. Such condition in the drinking water supply system can occur if the water is saturated with ${\text{CO}}_{3}^{2 - }$ and it is commonly found in the outer surface of the drinking pipes [1–3]. However, in the reaction with the hydrated products, acid is produced and thus the calcium from the hydrated products is leached out and the pH of the binder starts to drop. During carbonation, ${\text{CO}}_{2}$ penetrates through the porous binder material and react with the hydrates by leaching the calcium. Furthermore, subsequent reactions will enhance the mechanical properties of the structure 2, [4]. This will only occur in the internal surface if there is oversaturation of ${\text{CO}}_{3}^{2 - }$ compound and if the soil conditions allow carbon dioxide to be trapped near the surface of the pipe.

On the other hand, a cementitious structure in long term contact with water will leach out the free lime and calcium from the hydrated products of the cement. Specifically with low pH and low ion content water. This mechanism has been extensively reported and it decreases the strength of the structure. Largely because it is driven by diffusion of calcium towards the medium and no other compounds react with the hydrated products [5–7].

There are three methods currently available for inspection of cement-based pipes: acoustic, ground penetrating radar (GPR) and the phenolphthalein test. The acoustic method consists on monitoring the speed of sound waves traveling in longitudinal mode from two fixed points in the pipe section, providing information about the average state of the material between the measurement points. The GPR method consists in transmitting radio waves into the outer surface of the pipe. The radio waves are reflected/transmitted dependent on changes in material properties and used for inspection of sewage pipes mostly. For a correct inspection of the structure the pipes have to be dug out, which is costly and time-consuming. Similarly, the phenolphthalein test requires the pipes to be dig out and it is a destructive testing. Having possible hazardous components inside makes this test even more complicated.

The objective of this research is to detect anything from early degraded levels to full degradation in a mortar specimens from two chemical deterioration mechanisms: calcium leaching and acidic attack. Secondly, in this research a methodology is presented which determines the amount of degradation by pulse-velocity measurements through the thickness of the thin layered mortar. In this research measurements were performed on mortar specimens under laboratory conditions; no drinking water pipes were studied. Yet, this research is driven by stepping forward to the development of a non-destructive evaluation methodology that can potentially be implemented in cementitious (without ferrous components) drinking water pipes.

The material state of the water mains can be detected by means of ultrasonic non-destructive evaluation (NDE). The long term interaction of the pipes with the conveyed water and surrounding soil induces deterioration on both surfaces of cementitious pipes. The ultrasonic signature of this layers can be found with the ultrasonic pulse-echo (UPE) technique. Demčenko et al. [8] demonstrated that this technique is able to detect degradation due to acidic attack, based on finding a reflection from the healthy-degraded interface. Nevertheless, this method is limited by the resolution of the degraded depth reflection. Increasing the measurement frequency (1 MHz) improves the resolution near interfaces, but at the cost of higher signal attenuation. On the contrary, a larger wavelength (from 500 kHz frequency pump wave) will compromise near surface resolution but will increase the signal-to-noise ratio from the back wall reflection. Moreover, detection of chemical deterioration has been demonstrated by monitoring the change in speed of sound [9, 10].

Low frequency ultrasonic testing (50–500 kHz) has been extensively performed in cement-based structures [10–15]. Some of these techniques are pulse-echo and ultrasonic wave diffusion. Diffusion of ultrasonic waves have been used to measure crack depth in concrete with a range of 400–600 kHz [11]. This technique has been used likewise to characterize the dissipation and diffusion coefficients from ultrasonic waves in concrete structures [12] with the highest frequency at 800 kHz. Furthermore, mechanical properties from early stage setting of cement have been investigated by pulse velocity measurements [13]. Compressive damage and porosity estimation have been investigated by means of measuring speed of sound in the structure [10, 14, 15]. Recent studies have shown the possibility to inspect pipes with guided-waves. The focus has been made primarily in oil and gas pipes [16–18]. A study on early age properties of cementitious structures with guided waves was found based on pulse velocity and attenuation measurements as well [19]. Damage in cementitious structures such as cracks and inclusions were investigated as well with the wave guide technique [20, 21]. The early age of cement has also been of great interest for the scientific world. Methods such as pulse velocity and the ultrasonic wave reflection have been widely used characterize early stage properties of cement [22]. Guided wave testing is a very interesting method for large range applications e.g. pipes. However, the thickness of cement based pipes are not suited for such a technique as the wall thicknesses are out of the range of this kind of waves.

The formation of cementitious structures is mainly due to the hydration of cement. Production of Portland cement typically comes from the decomposition of limestone (calcium carbonate) and the addition of m-kaolin in a process called clinkering which then generates new compounds [23]. The main phases present in Portland cement after clinkering are: tricalcium silicate or Alite (3CaO.SiO_2_ or C3S), dicalcium silicate or Belite (2CaO.SiO_2_ or C2S), calcium alumino ferrite (4CaO.Al_2_O_3_Fe_2_O_3_ or C4AF) and tricalcium aluminate C3 A-alkali solid solution (3CaO.Al2O3 or C3A). The content of each phase in the total mixture can be controlled by the processing conditions as well as the mole fraction of m-kaolin to calcium oxide. In practice, the phases that provide higher strength and durability to the cementitious structures are approximately 45% and 25% of Alite and Belite respectively [23]. The resultant components of hydrating the main phases are the calcium silicate hydrates, better known as CSH gels, and the Portlandite or better known as calcium hydroxide. These two components contribute to the major part of the structural capacity. The hydration of the other phases will form lower amount of CSH and Portlandite but mainly will form Ettringite (depending on the Gypsum content) and mono-sulphoaluminate hydrates [23, 24].

**Fig. 1:**
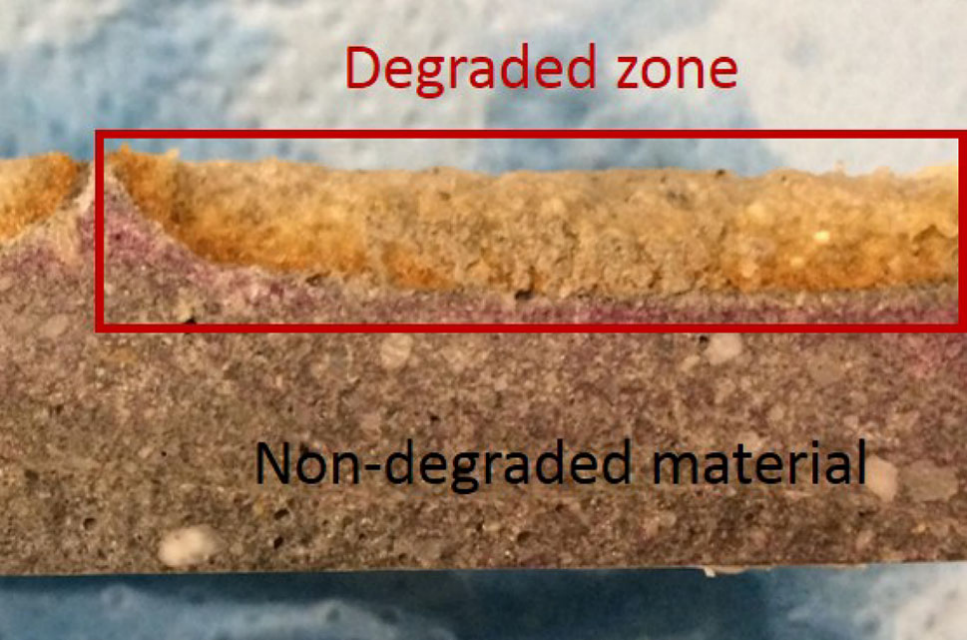
Acidic deterioration in cementitious material. Cross-section of specimen subjected to degradation from only one side. Sharp transition between pristine and degraded material

An acidic environment can be very harmful for cementitious structures. Moreover, the acid can dissolve and crumble completely the binder material leaving no structural strength. Wang et al. [1] reported that it is highly unlikely that the supplied drinking water has a low pH and thus no contact with an acidic environment would be expected. Nonetheless, the generation of bacteria layer (biofouling) in the internal surface is of acidic nature. It has been shown that this layer will attack the structure in an acidic manner [1–4]. The contact with acidic environment dissolves the Calcium content present in the structure. Thus, the main hydrate compounds (calcium hydroxide and calcium silicate hydrates) are leached and dissolved. The time at which these reactions occur is dependent on the acidity of the solution. The rest of the hydrated products are decomposed [25–27] leading to material crumble. Figure 1 depicts a cross-section of a cementitious structure subjected to one-sided (upper surface) acidic deterioration.

A visual change in the structure can be seen between degraded and non-degraded material (see Fig. 1). Further, in the degraded zone, a thin brownish coloured layer can be seen at the edge of the degraded zone. This ferric hydroxide layer accumulates the salts that are being removed from the binder. The later was reported by Chandra [25] where she concluded that just after the brown ring, the calcium content completely drops due to dissolution. The ferric hydroxide layer remains with a constant thickness. In the degraded zone there is no binder anymore and the remaining compounds and aggregates easily crumble.

Dissolution of Portlandite is the dominant reaction during contact with low ion content water [28]. The followed reacting compounds to the Portlandite are the monosulfoaluminate, ettringite and the progressive dissolution of the CSH gels [25–27]. Thus, leaching of calcium does not dramatically reduce the strength of the structure as compared to acidic deterioration. The calcium content in the material is equal to the calcium content in equilibrium state before the solid is in contact with water. The dissolution of Portlandite begins when the material is placed in contact with low-ion content water. The pH of the solution is dependent on the minerals present in the water. For instance, studies have shown that the measured pH from a drinking water supply system is 6.9 [28]. In another study, the recommended pH level in the drinking water is 8.2 ± 0.1 [29]. However, in a drinking water supply system, the measured pH is directly dependent on the location.

From the transport law of diffusion and dissolution, the mass balance equations are

1 $$ \frac{{\partial \left( {\emptyset \cdot C_{Ca} } \right)}}{\partial t} + \frac{{\partial S_{Ca} }}{\partial t} = \nabla \cdot \left( {D \cdot \nabla C_{Ca} } \right), $$

where $C_{Ca}$ is the calcium concentration in the pore solution, $S_{Ca}$ is the real mass density of the solute, $D$ is the diffusivity of calcium in the pore solution, $\emptyset$ is a factor related to the porosity in the solid, $\nabla C_{Ca}$ is the concentration gradient [33–35]. This is a complex phenomenon that has been extensively studied [30, 31]. One direct change due to the local decrease in mass of the solid is the local density decrease.

**Fig. 2:**
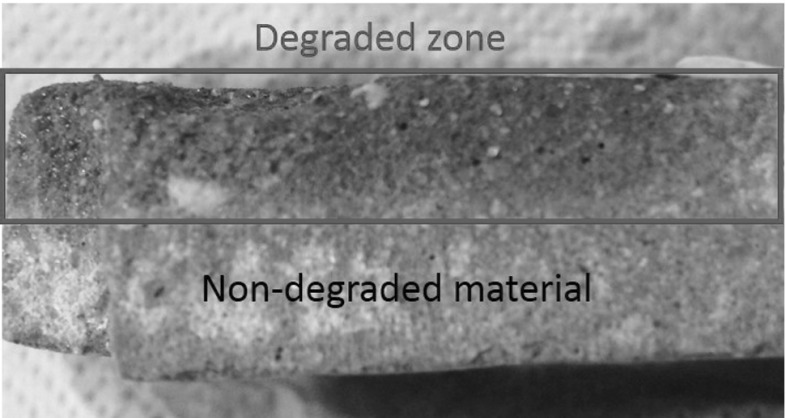
Leaching of calcium in cementitious material. Transition between pristine and degraded material is composed of gradual layers of calcium content (Color figure online)

Figure 2 depicts the degradation profile of a specimen’s cross-section. The part in the red box shows the material that was subjected to leaching. Leaching in this case originated from the top surface. There is no clear sharp transition nor difference in the material structure. The degraded material in this case has much higher remaining strength capacity than compared to a material deteriorated by acid.

The decrease in Calcium content as shown in Eq. 1 has a direct effect in the mass of the solid and thus a direct effect on the density. Local densities will change as degradation penetrates the solid which in turn changes the acoustic velocity. The speed of sound in a solid medium is defined as

2 $$ V = \sqrt {\frac{{E\left( {1 - \nu } \right)}}{{\rho \left( {1 + \nu } \right)\left( {1 - 2\nu } \right)}}}, $$

where $E$ is the Young’s modulus and $\nu$ is the Poisson’s ratio.

**Fig. 3:**
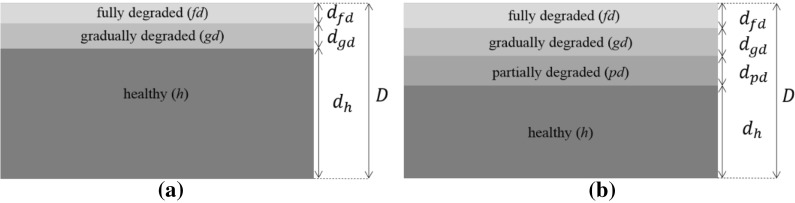
Leached cementitious structure **a** with low permeability and degradation resistant and **b** with high permeability and non-degradation resistant admixtures

The degraded zone consists of two layers if the cementitious structure has very low permeability or if the properties of the cementitious structure are leaching resistant [30–32, 34, 35]: a fully leached zone and a partially degraded zone with a gradient of Calcium content (see Fig. 3a). The intermediate layer has a negligible thickness compared to the pristine and fully degraded thicknesses. The latter has only decalcification of the calcium silicate hydrates. On the contrary, if no admixtures are included during the setting of the cement or the cement has high permeability, the leached layer is composed of three internal layers (see Fig. 3a). The two intermediate layers have a comparable thickness with respect to the pristine and fully degraded thicknesses [30–32, 34, 35]. The gradually degraded layer has a gradient of calcium content from the calcium hydroxide dissolution and decalcification of the CSH. The partially degraded layer has only dissolution of calcium hydroxide. The density has a direct effect due to the calcium content in the different layers and thus the acoustic properties as well.

The layered structure defined in Fig. 3 is a function of the calcium content, thus Fig. 3a and b are two different cases of calcium leaching. For acidic attack only Fig. 3a represents the calcium content distribution. Experimental evidence of the calcium distribution along the thickness, the mechanical strength and the porosity can be found in the literature [24–27, 30–35]. This corroborates the distribution of the layers shown in Fig. 3a and b.

## Methods

In order to characterize the response of the ultrasonic waves to the long term degradation of cement-based pipes in a laboratory scale the following steps are proposed: (1) production of mortar specimens and preparation; (2) accelerated degradation of specimens; (3) ultrasonic test; (4) signal processing; (5) estimation of degraded depth; and (6) destructive testing.

As mentioned earlier, degradation in cementitious drinking water pipes takes place from the inner and outer surface. The exposure time intervals are explained in the following section. It is important to note that the same procedure was followed for each degradation mechanism separately. Thus, the specimens submerged in one solution were not submerged in the other solution. In this way there was no interaction between the two degradation mechanisms. Finally, the effect of the material composition on the response of the ultrasonic signals to degradation was investigated by manufacturing two different batches with different material composition.

### Production of Specimens and Preparation

Mortar blocks with thickness of 20 mm were manufactured with Portland cement (CEM I). The recipe of the manufactured specimens is shown in Table 1. A coating was placed on the back of the specimens and in between the areas to be degraded in order to obtain many degraded states in one specimen and to avoid the double sided degradation for the one-sided degradation tests. The specimens were kept immersed in water after the curing time, during storage and during the ultrasonic tests. Water saturation was kept during the experiments.

**Table 1:** Composition mortar specimens

w/c ratio	CEM I (kg)	Sand (kg)	Plasticizer (kg)	Water (kg)
0.3	0.827	1.274	0.021	0.247
0.4	0.718	1.274	0.0061	0.287

### Accelerated Degradation

The specimens from batch 1 were immersed in 6 molar ammonium nitrate (AN) solution and the specimens from batch 2 were immersed in 1% concentration hydrochloric acid (HCL) solution. The measured initial pH values are 5 for the ammonium nitrate solution and 1 for the hydrochloric acid solution. The degradation interval times for the one-sided experiments were divided in early stage with 1–7 days of exposure to the solution and long deterioration with, 21, 35, 49, 63, 77 and 91 days of exposure to the solution. For the two sided degradation the interval times were 7, 14 and 28 days. The solutions were renewed every 2 days in order to maintain a constant degradation rate. Acceleration of calcium leaching can be done by ammonium nitrate solution [9]. The reaction continues with the mono-sulfoaluminates, ettringite and finally to the progressive decalcification of the gels. The calcium leaching does not break down the gels but only gradually dissolves the calcium and thus the compound is still present. On the contrary the acid compound completely fragments the CSH gels.

### Ultrasonic Test

Dispersive behaviour has been reported extensively reported [8, 19–21]. Wave distortion as well as group velocity are the two main challenges found in ultrasonic testing in cement-based structures due to dispersion. Coarse grain structure, porosity and composite nature of the cement-based structures are the main contributors to this phenomenon. In similar studies Aggelis et al. [19] measured the attenuation, frequency response and dispersion of ultrasonic waves as a function of the sand content and water to cement ratio of the mortar specimens. He concluded that the main source of error is the detection of the time delay of the signals thus an average of many signals was used. The dispersive behaviour is very important to consider, however, dispersion of ultrasonic waves has negligible effect on the measurements performed due to the signal processing scheme presented. Furthermore, the literature shows that the dispersion of ultrasonic waves at the frequencies proposed in this research are minimal in comparison with the change in pulse velocity in a degraded mortar. Demčenko et al. [8] showed that there is no dispersion effects in a fully degraded mortar.

**Fig. 4:**
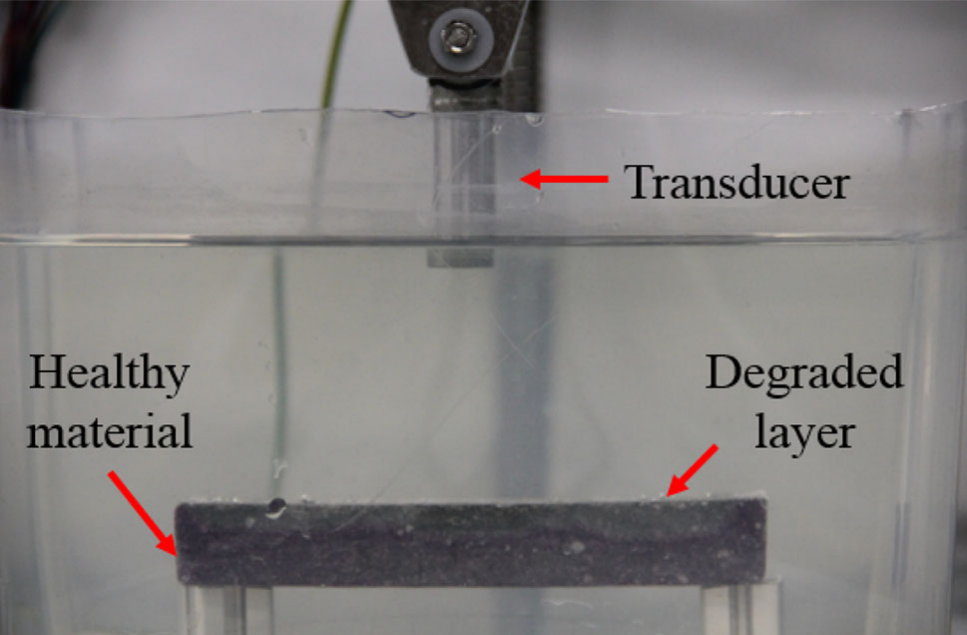
Immersion ultrasonic test set up with a degraded cementitious specimen

The specimens were placed in an immersion tank (free of degradation agents) as shown in Fig. 4. A uniform coupling between the transducer’s surface and the specimen was achieved. In an inline inspection, it is possible to access to the internal which is one of the main constraints. Therefore, pulse-echo is the most suitable technique to measure the speed of sound in the structure. The tests were performed on the deteriorated side (for the one-sided degradation). Two flat non-focused transducers with 0.5 MHz and 1 MHz central frequencies were used with 0.75″ and 0.5″ diameters respectively. The data was recorded with a 16 bits resolution digitizer at a 62.5 MS/s sample rate.

**Fig. 5:**
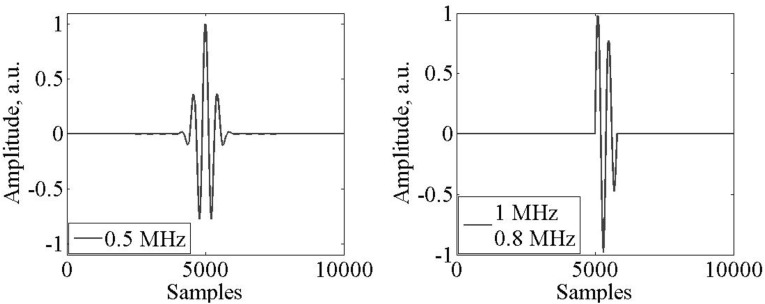
Input signals with **a** Windowed sine; **b** exponential decrease sine

The input voltage used is shown in Fig. 5. A windowed sinusoidal signal was applied to the 0.5 MHz transducer and a sinusoidal signal with exponential decrease was applied to the 1 MHz transducer. In the latter two different excitation frequencies were used: 0.8 MHz and 1 MHz. The response of the transducer to the second input signal further reduced the resonance of the piezoelectric element. The frequency components of such input signals was very important for the signal processing procedure. Each of the input signals has a specific set of frequency components and lastly the ringing of the transducer was reduced allowing spatial resolution of close reflections. On the contrary, spike-shaped excitation signals generate a long resonance response of the piezoelectric element as well as higher energy harmonics [10].

### Signal Processing

The effect of acoustic and electronic noise can be reduced by dedicated hardware components or software implementation. Digitally processing the data requires the analysis and the implementation of processing algorithms. Nevertheless, one of the main advantages of post-processing is that the parameters of interests can be accurately extracted without the need of calibration procedures. In this work, the main parameters to extract are the amplitude and time of arrival from the water-cement interface (front wall) and the cement–water interface (back wall). Thus, following scheme is proposed: (1) noise removal (by Wiener Filtering); (2) analytic envelope calculation.

#### Noise Removal

As mentioned, the Wiener filter is used for noise reduction and the removal of frequencies uncorrelated to the ultrasonic waves. This procedure enables to extract the specific frequency components to the signal of interest. The amplitude and time of arrival from the reflected signals are the main parameters for solving this formulation. However, a priori information has to be known from the scattering amplitudes. Thus the transfer function has a particular value for each reflected wave, in this case, the front wall reflection and the back wall reflection. The model presented by Neal et al. [36] is

3 $$ y\left( t \right) = h\left( t \right)*x\left( t \right) + n_{a} \left( t \right) + n_{e} \left( t \right), $$

where $y\left( t \right)$ is the signal recorded with the acquisition unit, $x\left( t \right)$ is the unknown impulse response function of the scattering amplitude, $h\left( t \right)$ is the transfer function of the system, $n_{a} \left( t \right)$ is the acoustic noise (porosity, impurities, internal interfaces) and $n_{e} \left( t \right)$ is the electronic noise. The transfer function $h\left( t \right)$ is the system response and it considers the voltage input to the sensor, the response from the electronics, the response from the transducer and the propagation effects of sound waves in the material (diffraction, attenuation, interface of mediums, etc.) [36–39]. The noise components can be grouped in a single term $n\left( t \right)$. Equation 3 is transformed into the frequency domain

4 $$ Y\left( \omega \right) = H\left( \omega \right)X\left( \omega \right) + N\left( \omega \right). $$

Solving for $X\left( \omega \right)$ directly does not give meaningful results due to the mathematical ill posed problem [36]. $X\left( \omega \right)$ is solved from a statistical point of view where $X\left( \omega \right)$ and $N\left( \omega \right)$ are uncorrelated Gaussian random variables with known variance and mean. Thus, $Y\left( \omega \right)$ is a random Gaussian random variable as well. The transfer function $H\left( \omega \right)$ is a non-random known value [36]. The estimated value of $X$ can be obtained from the maximum value of the probability density function $f\left( {X/Y} \right)$ if this is a Gaussian random variable. Details of the derivation can be found in literature [36]. The estimated value of *X* is obtained in the frequency domain

5 $$ \hat{X}\left( \omega \right) = \left[ {\frac{{H^{*} \left( \omega \right)}}{{\left| {H\left( \omega \right)} \right|^{2} + \sigma_{N}^{2} /\sigma_{X}^{2} }}} \right]Y\left( \omega \right). $$

The estimated value $\hat{X}\left( \omega \right)$ is then transformed to the time domain as $\hat{x}\left( t \right)$ [36–39].

#### Analytic Envelope (Hilbert Transform)

Accurate amplitude and time of arrival can be obtained from the extraction of the analytic envelope if noise has been removed [41–44]. In order to extract the analytic envelope, the square root of the sum of the squares of the real and imaginary parts are calculated for each point in time

6 $$ a\left( t \right) = \sqrt {\left( {x_{r} \left( t \right)} \right)^{2} + \left( {\frac{1}{\pi }\mathop \smallint \limits_{ - \infty }^{\infty } \frac{{x_{r} \left( {t^{\prime}} \right)}}{{\left( {t - t^{\prime}} \right)}}{\text{d}}t^{\prime}} \right)^{2} } ,$$

where $x_{r}$ is the real part of the signal and the second term is the Hilbert transform [40]. Finally, the time of arrival is extracted from the maximum peak from the respective reflections of interests, in this case the front wall and back wall reflections [40–44].

**Fig. 6:**
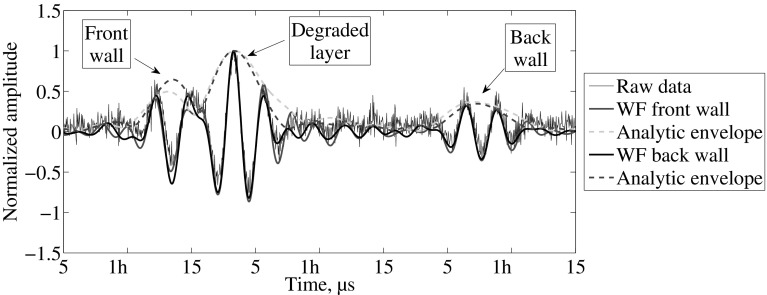
Typical data for acidic deterioration obtained after 3 days in one-sided acidic solution. The signal processing steps are shown as the Wiener filter and analytic envelope (Color figure online)

**Fig. 7:**
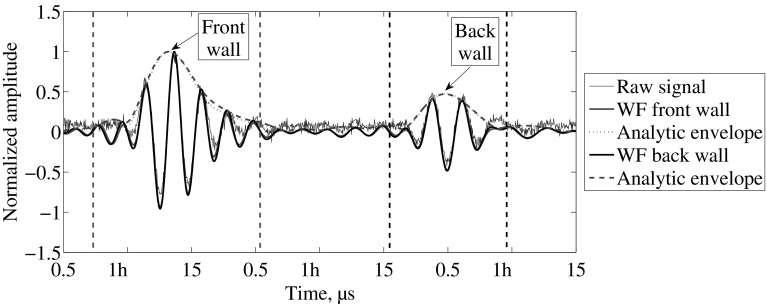
Typical data for calcium leaching obtained after 4 days in one-sided deterioration configuration. The signal processing steps are shown as the Wiener filter (WF) and analytic envelope (Color figure online)

The processing steps for the experiments performed in the mortar under acidic attack are depicted in Fig. 6. The blue line represents the raw signal, while the red and black lines represent the data after Wiener filter for the front wall reflection and back wall reflection respectively. The dotted line represents the analytical envelope of the filtered data. As mentioned in the previous section, the change in the material properties is sharp in an acidic deterioration environment. For this reason it is possible to see a reflection from the deteriorated layer. In the literature, the detection mechanism is based on such reflection [8]. Nevertheless, it was found that such reflection cannot be found in a calcium leaching scenario (see Fig. 7). Furthermore, Fig. 7 depicts the typical signal recorded from the experiments of a mortar degraded by contact with ammonium nitrate solution. The red lines and the black lines enclose the part of the signal that is used to obtain the frequency components and perform the Wiener filter. The signal-to-noise ratio clearly improves the detection of the reflection of interest.

### Estimation of Degraded Depth

The estimation of degraded depth from measuring the acoustic velocity is directly related to the degraded depth measured from the destructive test. One way to do this is to firstly characterize the speed of sound in pristine and fully degraded material. The total time that the waves take to travel through the structure is

7 $$ t_{D} = t_{h} + t_{pd} + t_{gd} + t_{fd}, $$

where the sub index $pd$ is partially degraded, $gd$ is gradually degraded and $fd$ is fully degraded. Then the distance over velocity is substituted:

8 $$ \frac{D}{{V_{D} }} = \frac{{d_{h} }}{{V_{h} }} + \frac{{d_{pd} }}{{V_{pd} }} + \frac{{d_{gd} }}{{V_{gd} }} + \frac{{d_{fd} }}{{V_{fd} }}, $$

the remaining healthy wall thickness is then substituted by

9 $$ d_{h} = D - d_{pd} - d_{gd} - d_{fd}, $$

the following relations are established in terms of the degraded depth and the speed of sound in healthy material

$$ \begin{aligned} & d_{pd} = ad_{d} ;\,d_{gd} = bd_{d} ;\,d_{fd} = cd_{d} ;\,V_{pd} = \alpha V_{h} ;\\& \,V_{gd} = \beta V_{h} ;\,V_{fd} = \gamma V_{h}\end{aligned} $$

where, $a$, $b$ and $c$ are the fraction of material with respect to the total degraded area and $\alpha$, $\beta$ and $\gamma$ are the fraction of speed of sound with respect to healthy material. Substituting these relations into Eq. 8 and solving for the degraded depth $d_{d}$ the following is obtained

10 $$ d_{d} = \frac{D}{{V_{D} }}\left( {\frac{{{V_{h}-{{V_D}} }}}{{\frac{a}{\alpha } +
\frac{b}{\beta } + \frac{c}{\gamma } - 1}}} \right), $$

where $V_{D}$ is the speed of the total thickness, $V_{h}$ is the speed of sound in pristine material and $D$ is the thickness of the specimen. From Eq. 10 the degraded depth can be predicted from the ultrasonic measurements. This relationship was corroborated with destructive testing.

The Eqs. 7–10 describe the speed of sound distribution in a layered medium. The equations do not take in consideration the propagation of sound waves in a solid elastic medium. Thus, in this scheme the dispersion, attenuation and absorption of sound waves are not included in the quantification of degradation. The interaction of reflections as well is not considered in the equations described in this section.

### Destructive Testing and Validation

In order to measure the degraded level, the specimens were cut in the middle of the degraded areas. For the calcium leaching case, the degraded depth cannot be seen directly. A Phenolphthalein solution was sprayed in the fresh cut surfaces. If the pH of the surface is higher than 8.2 then the solution remains purple, this is an indication of non-degraded material. On the contrary if the pH of the specimen’s surface is below 8.2 then the solution becomes colourless. This is an indication of degraded material. The distance between the specimen’s surfaces to the end of the colourless zone is the degraded depth. The degraded level was measured along the cross section of the specimen’s and mean and standard deviation values were obtained. For the case of acidic attack, the degraded depth is visible and thus no phenolphthalein solution was used. The predicted degraded depth based on Eq. 10 was validated with the measured degraded depth for each degradation mechanism.

## Results and Discussion

The effect of acidic deterioration and leaching in the ultrasonic response had a major discrepancy. A reflection from the degraded layer was found in the mortar deteriorated by acid [8, 10]. However, a detectable reflection from the degraded layer will not always be present, which is one of the major problems of this methods found in literature [8–10]. Thus, in this research the focus is on the reflections from both surfaces of the material. This is due to the width of the front wall signal as well as the low reflection coefficient from the back surface with respect to the front surface reflection. In this case, a slight increase in the degradation enabled to resolute both signals.

### Estimated Depth from Calcium Leaching

**Fig. 8:**
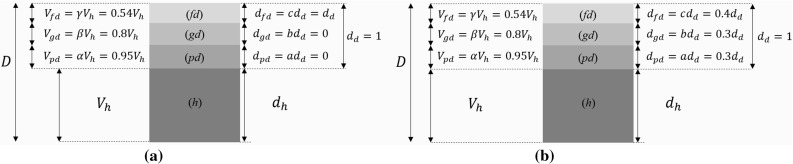
**a** Healthy and fully degraded configuration are present in the structure. **b** Degraded zone dominated by gradually and partially degraded regions. This is considered as a “worst” case where the leaching process is not as sharp and the properties of the material gradually extend from healthy to fully leached

Equation 10 was solved for two main scenarios. In the first case the two layered structure is considered with only pristine and fully degraded material. A second relevant situation is when the degraded thickness is dominated by the partially degraded and gradually degraded layers. These two scenarios are depicted in Fig. 8. The total degraded depth shown in the solution of Eq. 10 is $d_{d}$ only. Thus, the same Equation was solved as the degradation progress through the thickness of the mortar. The parameter γ is obtained by the ratio of the pulse velocity at full degradation and pulse velocity at pristine state. Then parameters *β* and *α* are set from the calculated γ in the pristine state. Furthermore, parameters *a* and *b* are assumed to be as large as possible (≈ 0.3) in the second scenario whereas in the first scenario these parameters are assumed to be as small as possible (≈ 0).

**Fig. 9:**
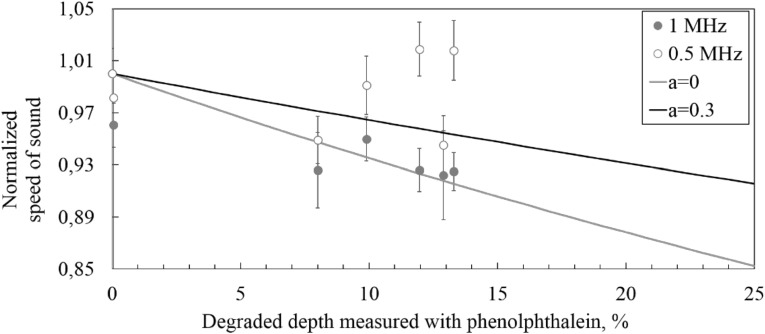
Speed of sound change from early stage degradation. Solid lines: model from Eq. 10 evaluated with $b = a$ and $c = 1 - a - b$; $\gamma = \left( {V_{fd} /V_{h} } \right)$

**Fig. 10:**
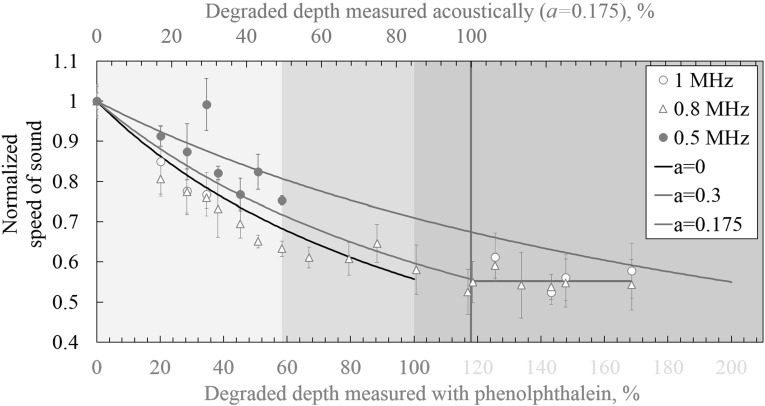
Speed of sound profile with 0.5, 0.8 and 1 MHz central frequency excitation signals (markers). Solid lines: model from Eq. 10. Light blue region: measurements with unmodified thickness. Middle blue zone: measurements with reduced thickness up to largest degraded depth. Dark blue zone: measurements from reduced thickness greater than the measured degraded depth (phenolphthalein tests) (Color figure online)

The change in the acoustic velocity of various degraded levels can be seen in Figs. 9 and 10 for early stage and long deterioration respectively. Only after testing with frequencies in between 0.5 and 1 MHz it was found that the standard measurement deviation for the pristine state is higher for tests performed with lower frequency compared to higher frequency. Additionally, for the lower frequency signals, an increase in the speed of sound in early degraded material was found (see Fig. 9). Both the high scatter and the increase in speed of sound are due to the resolution problem with low frequency signals. For higher frequency waves this problem does not exist.

In Figs. 9 and 10 these two cases are shown as *a* = 0 and *a* = 0.3. In this research these two conditions are considered as the boundaries of the possible leaching events that can happen in a cementitious structure.

Figure 10 shows the results for long term degradation with 0.5, 0.8 MHz and 1 MHz. The back wall reflection from the 1 MHz central frequency tests were heavily damped after 45% of degradation depth. The back wall reflection from the two other frequencies were heavily attenuated after 60% of degradation depth. In order to characterize larger degradation depths, the specimen’s thickness was gradually reduced from the healthy side towards the degraded side by dicing. Thus, instead of inducing further degradation the material is made thinner, resulting in an overall higher degradation. These results are shown in the two darker blue zones in Fig. 10. The degradation degree was obtained by dividing the measured degraded depth ($d_{d}$, from phenolphthalein tests) with the thickness of the specimen, *D*; $\left( {d_{d} /D} \right)*100\%$. The results from the ultrasonic tests were correlated with the phenolphthalein tests as shown in Fig. 10 bottom axis.

During this process, it was found that, if the thickness of the specimen was reduced to $d_{d} > D$, where $d_{d}$ is measured from the phenolphthalein tests, the speed of sound kept decreasing. An apparent degraded depth higher than 100% is shown in the bottom axis of Fig. 10. After 117% of apparent degraded depth, the speed of sound did not drop further. Thus, the fully degraded condition was reached. On the top axis, the degraded depth obtained from Eq. 10 when *a* = 0.175 shows a 100% degraded depth when the phenolphthalein results show 117% (bottom axis). This discrepancy is due to the layers present in the structure. While the phenolphthalein only shows a change in pH from a threshold value Eq. 10 shows the fully degraded condition.

### Validation of Estimated Depth from Acidic Deterioration

**Fig. 11:**
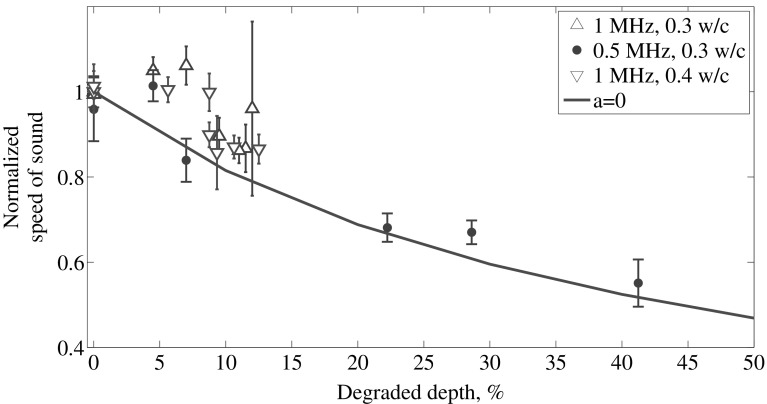
Speed of sound profile for the different degraded depths measured with 0.5 MHz and 1 MHz central frequency transducer. Solid line: model from Eq. 10

Figure 11 depicts the speed of sound profile for the different degradation depths. The measured speed of sound in fully degraded material due to acidic attack is found to be 1400 (m/s) with a 0.5 MHz central frequency actuation signal. This is corroborated with the literature [8, 10]. Due to the heavy attenuation of the waves, new batch of specimens with a lower water-to-cement ratio (0.3) were manufactured and degraded. Experimental results of the lower w/c cement content agree with model from Eq. 10 evaluated at *a* = 0.

**Fig. 12:**
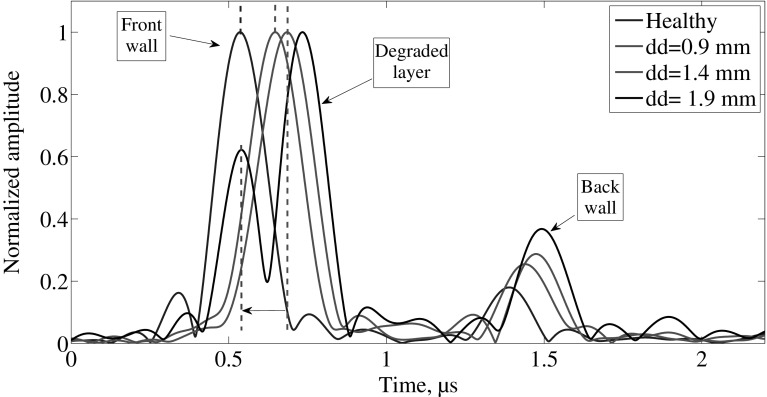
Early stage degradation signals from acidic deterioration. Doted lines represent the location in time of the front wall reflection. The data was normalized to the maximum of each signal. Thus it is not possible to see the reduction in amplitude from the front wall. In the legend, *dd* is degraded depth

The increase in the speed of sound in the early stage degradation is due to the proximity between the front wall reflection and the reflection from the degraded layer. In Fig. 12, the process where the front wall is shifted in time to the right as the degraded layer increases. Once the degraded layer is far enough from the front wall reflection, it is shifted back to the initial position as depicted by the dotted lines in the front wall reflection. Furthermore, the back wall reflection appears to increase in amplitude. This is due to the reflection coefficient decrease at the water-degraded interface due to the decomposition of the material.

### Response at Acoustic Interfaces

**Fig. 13:**
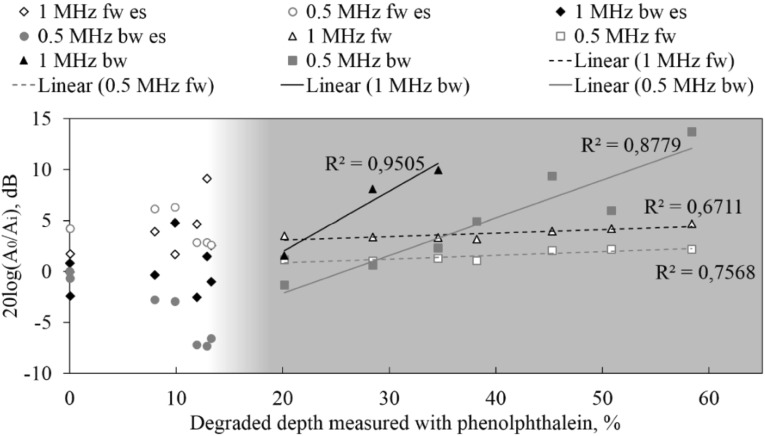
Front wall and back wall amplitude change (in dB). Trendlines are shown for the long term degradation. Gradual colour shows the change from early degradation (white) to long term degradation (blue). In the leyend, early stage is abreviated as (es), back wall is abreviated as (bw) and front wall is abreviated as (fw) (Color figure online)

The amplitude from the reflection at the water-degraded mortar interface (front wall) change as the degradation level increases. At very early stage there is high variation in the amplitude due to the incomplete degradation at the surface as shown in the white region from Fig. 13. At further degradation levels, the acoustic interface mismatch does not change as full degradation is present at the surface as depicted in the blue zone in Fig. 13. On the other hand, the amplitude of the mortar-water interface (back wall) does not change as the back side of the specimen remains healthy (non-degraded). Only when the degradation depth has penetrated the full thickness there will be an effect on the interface mismatch at the back of the specimen. The decrease of amplitude at the first interface (front wall) during early stage degradation means that more energy is transmitted to the material (see Fig. 13). This effect is frequency dependent. The higher the excitation frequency the higher the reduction in front wall and back wall amplitudes. In Fig. 13, a gradual transition between early degradation and long degradation is seen from the white zone to the blue zone. The amplitude of the pristine state for both front wall and back wall was considered as the reference for calculating the dB change shown in Fig. 13.

For the case of acidic deterioration, the attenuation increased as the degradation depth increased. In this case, in the early stage degradation there is no increase in the back wall reflection amplitude. The water/degraded reflection coefficient remains constant as soon as degradation is present in the material and this does not further change with respect to the degraded depth. The determination of the amount of degradation based on Eq. 10 had a similar response compared with the correlation between the acoustic and non-acoustic measurements. The speed of sound decreased as the degradation depth increased. In some cases, the experimental results showed an increase in pulse velocity, especially in the very early stage, and the decrease rate of speed of sound as the degraded depth increases was different.

There are many factors that are attributed to these discrepancies. For instance, in the early stage acidic attack, the proximity between the front wall and degraded layer reflections caused an apparent increase in the speed of sound. After 10% of degraded depth, Eq. 10 and the experimental results show a good agreement.

**Fig. 14:**
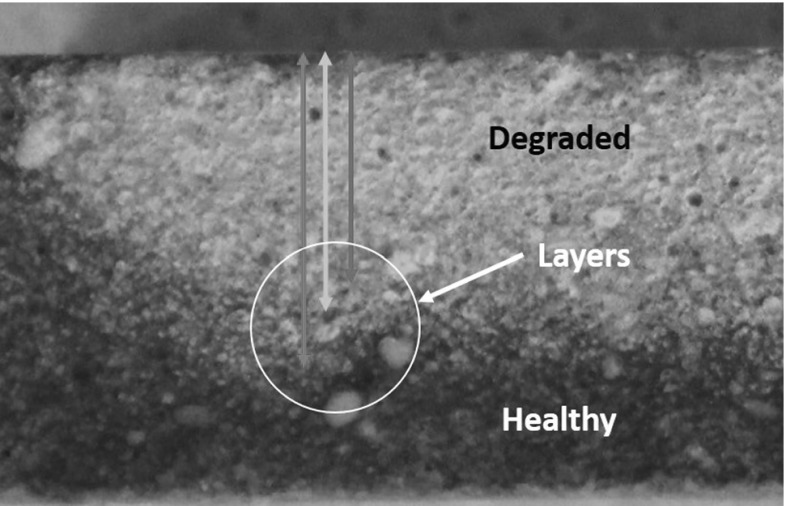
Cross-section view of a mortar degraded from contact with ammonium nitrate solution. Purple colour represents high pH (pristine). Grey zone represents low pH (degraded) (Color figure online)

The case of calcium leaching has a different story. This is mostly because the degraded zone is not as sharp as in the acidic deterioration (see Fig. 1). In Fig. 14, the transition between degraded and non-degraded is not very clear. The purple indicator gradually changes. In the experimental part, the degraded layer was considered until where the blue arrow in Fig. 14 is pointing. However, the identification from the purple coloration of the complete degraded zone was not possible to achieve. Thus, the results from the calcium leaching should have a higher degraded depth for the measured pulse velocity.

As mentioned in previous section. The Eq. 10 is a distribution of material properties (including speed of sound) and thicknesses of layers. However, the response of the reflections from a layered structure from a wave propagation approach are different. For instance the increase in pulse velocity from early acidic deterioration. The assumption that the distribution of the degraded layers starts from a sharp transition to an extended gradual change in the material properties is something to investigate as well. The distribution of the layers is a complex phenomenon which is dependent on many factors such as: porosity, cement ratio, manufacturing procedure, additives, geometry as well as the characteristics of the aggressive solution.

A final source of discrepancy between the experimental work and the solution of Eq. 10 is the determination of the time-delay of the reflections of interests. The error was more prominent in the long degradation from calcium leaching where the standard deviation increases. The back wall reflection was at the level of the noise, thus increasing the error in the detection of the time delay.

## Conclusion

The amount of degradation in a cementitious structure can be determined with the methodology presented for both types of degradation mechanisms. It is important that the pristine condition and the fully degraded state are known. In this research the long term degradation from drinking water pipes was represented by accelerated chemical deterioration in mortar specimens. The manufactured specimens had high porosity and low compaction which lead to high permeability. The degraded layer was then composed by the partially, gradually and fully degraded zones. Even though, there was a difference between the destructive and the acoustic method, this can be accounted for when the fully degraded material is characterized. With the Eq. 10 the amount of degraded material can be quantified. Accurate degradation depth quantification in very early stage (< 8%) may not be possible, however, detection of very early stage (< 5%) is possible as in difference with previous research. Despite the discrepancy found between the experimental work and the solution of Eq. 10 it is believed that the solution of the latter has more accuracy than the measured degraded depth from the phenolphthalein tests. Which in a practical application this is more beneficial avoiding destructive testing.

With a combination of back wall amplitude and pulse velocity it is possible to determine the degraded level in the structure. The amplitude of the reflected waves at the surfaces of the structure provides also important information for the early degradation level. Smaller measurement wavelengths are more sensitive to degradation compared to higher frequencies. Thus, the actuation frequency has to be selected depending on the initial thickness of the specimen. In this research, the most suitable excitation frequency for a 20 mm thickness structure was 0.8 MHz. This allowed to detect up to 60% degradation level. For very early degraded depth, higher frequencies may be used. In the methodology presented it is vital that the acoustic measurements are correlated with the real measured thickness in order to characterize the pulse velocity as a function of the degraded depth. But also, to determine whether the structure is dominated by a fully degraded layer or if it is composed gradual and partial change of the calcium content. This is a subject of further studies.
